# CD226 knockout alleviates high-fat diet induced obesity by suppressing proinflammatory macrophage phenotype

**DOI:** 10.1186/s12967-021-03150-4

**Published:** 2021-11-25

**Authors:** Jingchang Ma, Wei Hu, Dongliang Zhang, Jiangang Xie, Chujun Duan, Yitian Liu, Yuling Wang, Xuexue Xu, Kun Cheng, Boquan Jin, Yuan Zhang, Ran Zhuang

**Affiliations:** 1 Department of Immunology, Fourth Military Medical University, 169 West Changle Road, Xi’an, 710032 Shaanxi China; 2 Institute of Medical Research, Northwestern Polytechnical University, 127 West Youyi Road, Xi’an, 710072 Shaanxi China

**Keywords:** CD226, HFD, Obesity, Macrophage, Polarization

## Abstract

Obesity is associated with chronic low-grade inflammation, contributing to an increasing prevalence of chronic metabolic diseases, such as insulin resistance, non-alcoholic fatty liver disease (NALFD), and steatohepatitis. Macrophages are the predominant immune cells in adipose tissues. Adipose tissue macrophages (ATMs) would switch to pro-inflammatory M1 state during obesity, causing local and systemic inflammation. However, the regulatory mechanism of ATMs has not yet been well described within this process. Using a high-fat diet (HFD)–induced mouse obesity model, we found that the costimulatory molecule CD226 was highly expressed on ATMs and knockout (KO) of CD226 alleviated obesity caused by HFD. Loss of CD226 reduced the accumulation of ATMs and hindered macrophage M1 polarization, with lower serum proinflammatory cytokine levels. Furthermore, deficiency of CD226 on ATMs decreased the phosphorylation levels of VAV1, AKT, and FOXO1 and thereby upregulated PPAR-γ. Further administration of PPAR-γ inhibitor restored M1 phenotype in CD226KO ATMs. In summary, loss of CD226 alleviates the HFD-induced obesity and systemic inflammation through inhibition of the accumulation and M1 polarization of ATMs in which PPAR-γ-dependent signaling pathway is involved, suggesting that CD226 may be identified as a potential molecular target for the clinical treatment of obesity.

**Supplementary Information:**

The online version contains supplementary material available at 10.1186/s12967-021-03150-4.

## Introduction

Obesity is a chronic systemic inflammatory disorder characterized by the dysfunction of hypertrophied adipocytes and the accumulation of immune cells in adipose tissue [1]. The development of inflammation within adipose tissue contributes to proinflammatory status and metabolic dysfunction in the whole body, including type II diabetes mellitus, non-alcoholic fatty liver disease (NALFD), atherosclerosis, and ischemic cardiovascular disease [2].

Macrophages are the predominant immune cells in adipose tissue, and they accumulate in obese mice and humans [2, 3]. Macrophages are heterogeneous. They are classified to be classically (M1) or alternatively (M2) activated based on distinct patterns of gene expression and function [4]. It has been proposed that during obesity, macrophages in adipose tissue (ATMs) undergo a ‘phenotypic switch’ from an anti-inflammatory M2 phenotype to a pro-inflammatory M1 state, and this conversion was a key contributor to the emergence of the local and systemic inflammation of adipose tissues [5]. However, the molecular details underlying inflammatory responses and the regulatory mechanism of ATMs in obese adipose tissue remain uncertain.

CD226—also known as DNAX accessory molecule 1—is an important costimulatory receptor on T cells, NK cells, and monocytes/macrophages [6–9]. Previous studies regarding CD226 primarily focused on its regulation on the T and NK cells, while recently emerging reports believe that CD226 is also involved in the processes of macrophage polarization, migration, and other functions. It was studied that CD226 deletion improved post-infarction healing and cardiac function by favoring macrophage polarization towards reparative phenotype [10]. CD226 also played a costimulatory role in antigen presentation by small peritoneal macrophages [11]. However, since macrophages are highly heterogeneous in various tissue content, the regulation of CD226 on ATMs needs to be studied, especially considering that macrophages are the most abundant immune cells in adipose tissues. In the present study, we utilized CD226 knockout (KO) mice to observe the roles of CD226 deficiency in the obesity and its related systemic inflammation induced by a high-fat diet (HFD). We found that deficiency of CD226 alleviated obesity and inflammatory state via inhibition of the ATM accumulation and the proinflammatory phenotype of macrophages. The potential underlying mechanism involved in this regulation was the peroxisome proliferator-activated receptor gamma (PPAR-γ)-dependent signaling pathway.

## Materials and methods

### Clinical serum

For the detection of soluble CD226 in serum, 79 samples were randomly selected from the clinical laboratory of Tangdu Hospital of Fourth Military Medical University (48 females and 31 males). Additional file 6: Table S1 showed the basic detailed information for participants. Subjects with inflammatory disorders, metabolic related diseases, or anti-inflammatory drug use were excluded. According to the Chinese standard of body mass index (BMI), we divided the subjects into three groups: normal group (BMI: 18.5–23.9; 35 samples), overweight group (BMI: 24.0–27.9; 31 samples) and obese group (BMI: ≥ 28.0; 13 samples). All procedures were approved by the Tangdu Hospital ethics committee and conformed with the principles of Helsinki Declaration, and all participants provided written and/or oral informed consent. The sera were stored at − 20 °C until use.

### Mice

CD226KO mice on a C57BL/6 background were kindly provided by Professor Marco Colonna [12]. CD226KO mice were hybridized with C57BL/6 wildtype (WT) mice (the Animal Center of the Fourth Military Medical University, Shaanxi, China) for several generations. Homozygous CD226 KO mice and their WT littermates were then used for following experiments. The WT and CD226KO mice were fed with a standard chow that provided 10% of calories from fat and a HFD consisting of 60% fat for 16 weeks, respectively, beginning at the age of 5–6 weeks. We used inter-cage rotation procedure to mitigate the potential cage effects on the intestinal microbiota [13, 14]. All experiments using mice were performed according to the Guide for the Care and Use of Laboratory Animals (NIH, Bethesda, MD) and approved by the Institutional Animal Care and Use Committee of the Fourth Military Medical University.

### Materials

FCS antibodies, APC-CD226 (clone: 10E5, No: 17-2261-82) and PE-F4/80 (clone: BM8, No: 12-4801-82), PECy5-CD11c (clone: N418, No: 15-0114-81), FITC-F4/80 (clone: BM8, No: 11-4801-82), Fixable Viability Dye eFluor™ 506 (No: 65-0866-14) and MitoSOX™ Red mitochondrial superoxide indicator (No: M36008) were supplied by Thermo Fisher Scientific Inc. (Waltham, MA, USA). FCS antibodies, FITC-CD11c (clone: N418, No: 117306), AF700-CD45 (clone: 30-F11, No: 103128), FITC-CD226 (clone: 10E5, No: 128803), PE-CD226 (clone: 10E5, No: 128806), APC-CD155 (clone: TX56, No: 131510), and APC-F4/80 (clone: BM8, No: 123116) were purchased from BioLegend (San Diego, CA, USA). The chow (10% of calories derived from fat, No: MD12031) and food for the HFD groups (60% of calories derived from fat, No: MD12032) were purchased from Medicience Ltd. (Yangzhou, Jiangsu, China). The triglyceride assay kit (No: C061), low-density lipoprotein cholesterol (LDL-C) assay kit (No: C063), high-density lipoprotein cholesterol (HDL-C) assay kit (No: C063), total cholesterol assay kit (No: C063), alanine aminotransferase (ALT) assay kit (No: C052), aspartate aminotransferase (AST) assay kit (No: C072), gamma-glutamyltransferase (γ-GT) assay kit (No: C009), alkaline phosphatase (ALP) assay kit (No: C003), and glycosylated serum protein assay kit (No: C024) were purchased from Changchun Huili Biotech Co., Ltd. (Changchun, Liaoning, China). The following antibodies were used for western blotting and immunofluorescence: anti-PPAR-γ mAb (clone: C26H12, No: 2435), anti-AKT mAb (clone: 40D4, No: 2920), anti-p-AKT Ser473 mAb (clone: D9E, No: 4060), anti-p-FOXO1 Ser256 mAb (clone: E1F7T, No: 84192), and anti-perilipin-1 mAb (clone: D1D8, No: 9349), purchased from Cell Signaling Technology (Danvers, MA, USA); anti-p-VAV1 Tyr174 mAb (clone: 28.Tyr 174, No: sc-135785), anti-VAV1 mAb (clone: D-7, No: sc-8039), anti-FOXO1 mAb (clone: C-9, No: sc-374427), and anti-β-ACTIN mAb (clone: 2Q1055, No: sc-58673), purchased from Santa Cruz Biotechnology Inc. (Dallas, TX, USA); anti-F4/80 mAb (clone: CI:A3-1, No: ab6640), purchased from Abcam Inc. (Cambridge, Cambridgeshire, UK); and CD226 pAb (No: 50232-M08H), purchased from Sino Biological Inc. (Beijing, China). Functional grade CD226 monoclonal antibody (clone: 10E5) was provided by Thermo Fisher Scientific Inc. The PPAR-γ-selective inhibitor GW9662 (No: S2915) was supplied by Selleck Chemicals (Houston, TX, USA). The secondary immunofluorescence antibodies used were as follows: CY3-goat anti-rabbit antibody (No: BA1032), supplied by Boster Biological Technology Co., Ltd. (Wuhan, Hubei, China); and Alexa Fluor 488-goat anti-rabbit antibody (No: GB25303) and CY3-goat anti-rat antibody (No: GB21302), supplied by Wuhan Servicebio Technology Co., Ltd. (Wuhan, Hubei, China). Collagen type I (No: DY40127) was purchased from Diyi Biotechnology Inc. (Shanghai, China), and lipopolysaccharide (LPS) (No: L2630) was purchased from Sigma-Aldrich Inc. (St. Louis, MS, USA).

### Sandwich ELISA

The anti-human CD226 monoclonal antibodies (mAbs) and ELISA kit were used as previously described [15]. Briefly, 100 μl of anti-human CD226 mAb (10 μg/ml in 0.05 M sodium carbonate buffer, pH 9.5) was added to each well of a Nunc Maxisorp ELISA plate (Nunc, Rochester, NY, USA) and incubated overnight at 4 °C. After a 3 × wash, plasma samples or standard CD226 recombinant protein serially diluted with PBS containing 0.1% BSA and 0.1% Tween-20 were added to the wells and incubated for 1 h at 37 °C. After extensive washing with PBS containing 0.1% Tween-20 (PBST), the wells were incubated with another anti-human CD226 mAb conjugated with biotin for 1 h at 37 °C. Then, 100 μl of commercial streptavidin–horseradish peroxidase (HRP) was added and color development was performed on a TMB visualized system. 450 nm absorbance was determined with a microplate reader (Bio-Rad, CA, USA).

### Histology

After feeding a HFD for 16 weeks as indicated, epididymal adipose tissues and livers were harvested from mice and dissected. The tissues were fixed in 4% buffered formalin overnight, then embedded in paraffin and cut into sections 4 μm in thickness. Following standard procedures, the sections were stained with Hematoxylin and eosin (H&E) to determine morphological changes according to our previous study [16]. For Oil Red O (ORO) staining, the tissues were embedded in OCT medium after fixing in 4% buffered formalin overnight. Then, the tissues were cut into Sects. 10 μm in thickness. ORO staining was performed according to a previous study [17].

### Flow cytometry

Epididymal fat tissues and spleens were harvested from mice after 16 weeks of HFD. After shearing with ophthalmic scissors, the epididymal fat was collected in a centrifuge tube and digested with collagen type I at 37 °C for 15 min. Triple fetal bovine serum (FBS)-containing medium was used for neutralization. The cell suspension was passed through a 40 μm cell strainer. The stromal vascular fraction (SVF) of the epididymal fat was obtained after centrifuging. Splenocytes were treated with NH_4_Cl buffer for 5 min to lyse the red blood cells. Flow cytometry staining was performed according to our previous study [16]. Briefly, prior to surface staining with antibodies, Fc-gamma receptors were blocked by incubating cells with anti-CD16/CD32 antibodies (eBioscience, 14-0161-82). Thereafter, cells were incubated with the appropriate primary antibodies diluted in FACS buffer (DPBS + 2% FCS) for 30 min at 4 ℃. Cells were analyzed with a NovoCyte flow cytometer (ACEA Bioscience). Data were analyzed using NovoExpress software (ACEA Bioscience).

### Immunofluorescence

Epididymal adipose tissue was obtained from mice fed with a HFD or chow at 16 weeks as indicated. The tissues were first fixed with 4% buffered formalin overnight, and then embedded in paraffin and cut into 4-μm thick sections. Following standard deparaffinization procedures as described previously [18], these sections were blocked with 3% BSA plus 0.1% Triton-X100 for 30 min at room temperature, then incubated with primary antibodies, anti-mouse F4/80, anti-mouse CD226, and anti-mouse perilipin-1 for 1 h at room temperature, and finally stained with the corresponding immunofluorescence secondary antibodies and DAPI. Images were obtained using a confocal microscope (LSM 800, Zeiss, Germany).

### Metabolic phenotype assays

To analyze the glycolytic rate or metabolic phenotype, the real-time extracellular acidification rate of CD226KO and WT ATMs was recorded by an XFp analyzer provided by Agilent Technologies Inc. (Paloalto, CA, USA). ATMs were seeded into an XFp cell culture plate at a density of 3 × 10^4^ cells/well in 80 μL RPMI1640 medium (containing 10% FBS and 1% penicillin–streptomycin). Following adhesion for 24 h, the medium was replaced with 180 μL correspondingly Seahorse-DMEM medium (containing 0.5% BSA and 2 mM glutamine). After machine testing, 10 mM glucose, 1 μM oligomycin, and 50 mM 2-deoxy-glucose were sequentially added to the media for analysis and recording.

### Dual-luciferase reporter assay

RAW264.7 cells were seeded in a 24-well plate in RPMI1640 medium containing 10% FBS without penicillin–streptomycin for adhesion. Then, the medium was changed to Opti-MEM, and the cells were starved for 1 h. According to the manufacturer's instructions, RAW264.7 cells were transfected with pGL3-PPAR-γ promoter or pGL3-basic plasmids by Lipofectamine 3000. pGL3-basic plasmids and pGL3-PPAR-γ promoter, which contain firefly and Renilla luciferase genes, were constructed by TsingKe. (Beijing, China). After an additional 24 h, 5 μg/ml 10E5 functional antibody (incubated and bound on protein G beads) or isotype antibody was added to stimulate the transfected RAW264.7 cells for another 24 h. The cells were first lysed using cell lysis solution provided by Beyotime Biotechnology Inc. (Shanghai, China). Firefly fluorescein enzyme and Renilla luciferase activity were then sequentially measured according to the manufacturer's instructions. Normalization of the relative light unit of firefly fluorescein to that of Renilla fluorescein was conducted to control for cell number and protein content variation. Finally, the relative light unit ratio was calculated.

### Immunoblot analysis

The cells were collected and lysed with RIPA buffer containing 1 mM phenylmethylsulfonyl fluoride and protease and phosphatase inhibitors (Roche Applied Science). Lysates were centrifuged at 10,000 g for 10 min at 4 °C. The supernatant was collected and its protein concentration was measured using the DC protein assay kit (5000116, Bio-rad). First, 50 μg of total protein was loaded in each well of the gradient gel. Second, proteins were transferred onto a PVDF membrane (Bio-rad, 170-4156) with a semidry system and subsequently blocked for 1 h at room temperature with 5% milk in 0.1% TBS-Tween. Third, the membranes were incubated overnight at 4 °C with primary antibodies. The appropriate HRP-linked secondary antibodies were used for chemiluminescent detection of proteins. Final, the membranes were scanned with a Chemidoc imaging system (Bio-rad) and quantified using Image Lab 6 software (Bio-rad).

### Cell culture

The SVF was isolated from the epididymal fat tissue using sterile collagen type I and then cultured in RPMI1640 medium containing 10% FBS and 1% penicillin–streptomycin. Following adhesion for 24 h, the supernatant was removed and discarded, then the ATMs were obtained. Peritoneal macrophages were isolated using PBS. Briefly, mice were euthanized with isoflurane, then precooled PBS was injected intraperitoneally and used to lavage the peritoneal macrophages. After seeding in a 24-well plate, the peritoneal macrophages were purified by discarding the suspended cells. Finally, the purified peritoneal macrophages were stimulated with or without 100 ng/ml LPS for 12 h, and quantitative real-time PCR (qPCR) was further performed to measure the expression levels of target genes. For the PPAR-γ blocking experiment, RAW264.7 cells were pre-treated with or without GW9662 (10 μM) for 24 h, then incubated with 100 ng/mL LPS for 12 h. Finally, flow cytometry analysis and qPCR were performed to examine the polarization of macrophages.

### Quantitative real-time PCR

Total RNA from the aforementioned tissues and cells was isolated with TRIzol Reagent (Invitrogen, Carlsbad, CA, USA). Then, 1 μg total RNA was reverse transcribed into cDNA by PrimeScript™ II 1st Strand cDNA Synthesis (TaKaRa, Dalian, Liaoning, China). Finally, qPCR was performed using SYBR Green as the detection system (TaKaRa) in a CFX96 Real-Time PCR System (Bio-Rad, Hercules, CA, USA). The sequences of the qPCR primers used are summarized in Additional file 6: Table S2.

### Statistics

Results were presented as mean ± standard error (SEM). A two-tailed, unpaired Student's *t*-test (for comparison within two groups) or Kruskal–Wallis test with Dunn’s multiple test (for score related or clinical data comparison) or one way ANOVA with Tukey’s multiple test (for comparison within three or more groups) was performed using GraphPad Prism software (San Diego, CA, USA). A *P*-value less than 0.05 was considered statistically significant (*P < 0.05, **P < 0.01). Three independent experiments were performed and each contained 3 replicates.

## Results

### HFD increases CD226 expression on ATMs

**Fig. 1:**
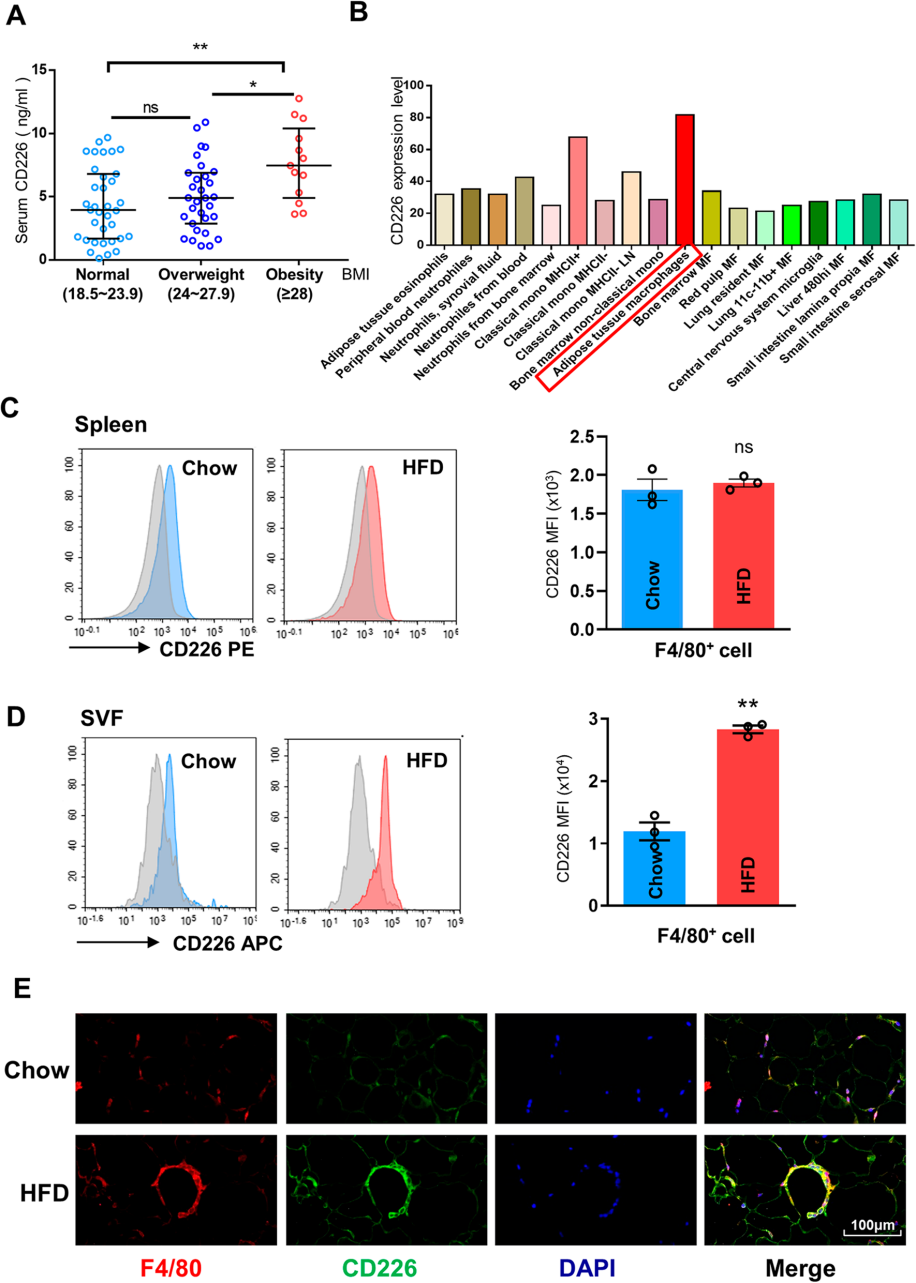
HFD increased CD226 expression in ATMs. **A** Comparison of soluble CD226 levels in sera of normal, overweight and obese individuals. Plots show individual data values obtained (79 individuals in total). Horizontal black lines represent median ± IQR. Differences between groups were determined by Kruskal–Wallis test. **B** The expression profiles of CD226 on myeloid cells, including monocytes, macrophages, and neutrophils. **C**, **D** FACS analysis of CD226 expression on macrophages in the spleen and epididymal SVF of C57BL mice fed with chow or a HFD for 16 weeks (*n* = 3). **E** Immunofluorescence staining for F4/80 (red) and CD226 (green) in the epididymal fat of mice fed with chow or a HFD for 16 weeks (*n* = 3). Scale bar means 100 μm. Data represent mean ± SEM. Differences between groups were determined by two-tailed, unpaired Student’s *t*-test. *P < 0.05, **P < 0.01

We examined the serum concentration of soluble CD226 in 79 human clinical samples (Normal 35, Overweight 31, Obesity 13), and found that the concentration of serum CD226 was significantly higher in the obesity cohort (median: 7.47 ng/ml, interquartile range [IQR]: 5.33–9.60 ng/ml) compared with the both normal (median: 3.96 ng/ml, IQR: 1.76–6.69 ng/ml) and overweight (median: 4.90 ng/ml, IQR: 3.10–6.75 ng/ml) groups (Fig. 1A), suggesting that CD226 might be related to the occurrence of obesity. Besides, the expression profiles of CD226 on myeloid cells (including monocytes, macrophages, and neutrophils) from immunological consortium ImmGen (Immunological Genome Project) [19] showed that CD226 expression on ATMs was higher than that on others (Fig. 1B).

Tissue macrophages are phenotypically heterogeneous. Through an obese mouse model, although the expression of CD226 was comparable between F4/80^+^ splenic macrophages from HFD-fed mice and those from chow-fed mice, its expression on F4/80^+^ ATMs from epididymal SVF of HFD-fed mice was significantly higher than that of chow-fed mice (Fig. 1C, D). Epididymal fat and SVF samples of HFD-fed mice also showed significantly increased *Cd226* mRNA levels than chow-fed mice (Additional file 1: Fig. S1A). However, HFD did not much alter the expression levels of CD226 on CD4^+^, CD8^+^, and NK cells in epididymal adipose tissues (Additional file 1: Fig. S1B). We further detected the expression of CD155 (a ligand of CD226) in ATMs derived from HFD-fed mice, which was also significantly higher (Additional file 1: Fig. S1C). Immunofluorescence staining of epididymal fat tissues showed crown-like structures of ATMs in HFD-fed mice, with CD226 robustly expressing on these crown-like structures (Fig. 1E). Moreover, CD226 was also upregulated in liver F4/80^+^ cells from HFD-fed mice and in RAW264.7 cells following LPS stimulation (Additional file 1: Fig. S1D, S1E). These data indicate a potential role of CD226 in ATMs of HFD-induced obese mice.

### CD226KO ameliorates HFD-induced obesity

**Fig. 2:**
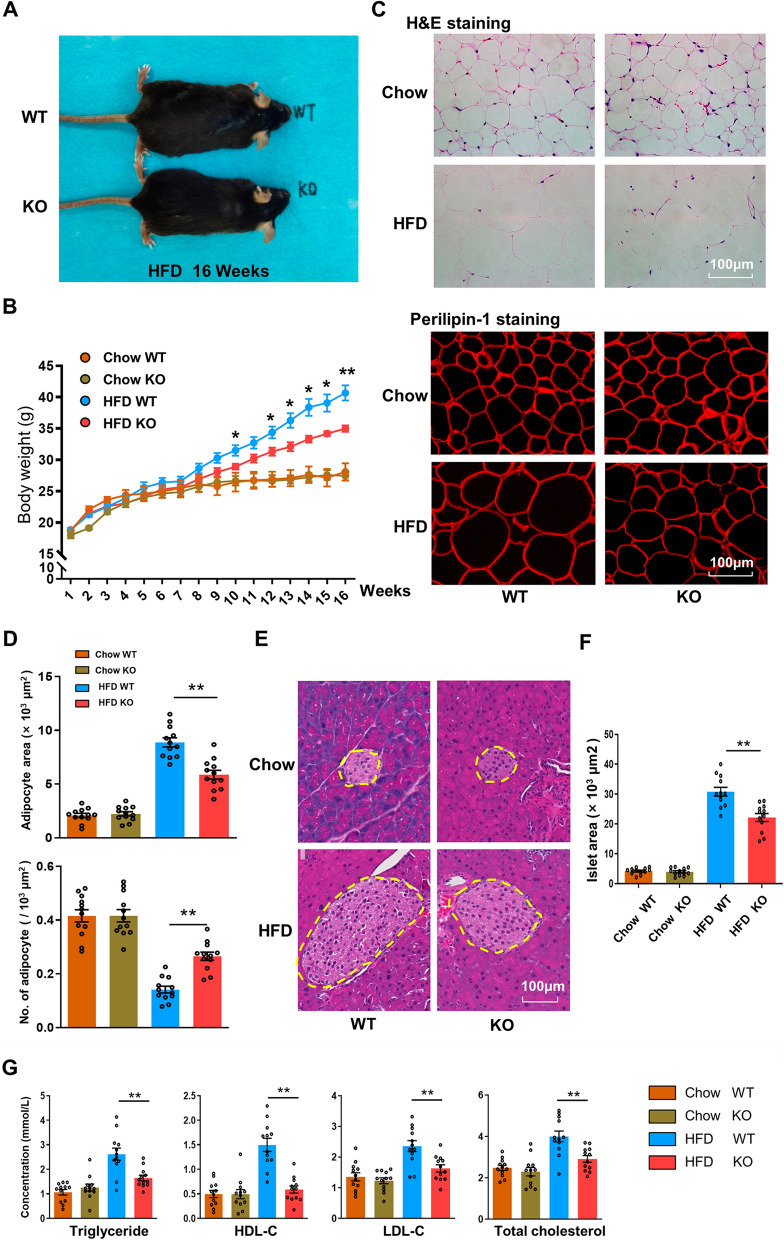
Obesity was ameliorated in HFD-fed CD226KO mice. **A** Representative images of WT and CD226KO mice fed with a HFD for 16 weeks (*n* = 6). **B** Bodyweight of WT and CD226KO mice fed with either chow or a HFD for 16 weeks (n = 4). **C** Representative H&E staining and perilipin-1 immunofluorescence staining of epididymal fat of WT and CD226KO mice fed with either chow or a HFD for 16 weeks (n = 4). Scale bar means 100 μm. **D** Adipocyte size and measurements of epididymal fat of WT and CD226KO mice fed with either chow or a HFD for 16 weeks. (n = 4). **E**, **F** Representative H&E images and islet size measurement of pancreatic tissue of WT and CD226KO mice fed with either chow or a HFD for 16 weeks. Scale bar means 100 μm. **G** Serum concentrations of triglyceride, HDL-C, LDL-C, and total cholesterol (n = 4). Graphs or combined data from three independent experiments. Data represent mean ± SEM. Differences between groups were determined by one way ANOVA with Tukey’s multiple test. *P < 0.05, **P < 0.01

To determine whether CD226 is involved in the occurrence of HFD-induced obesity, we examined the metabolic phenotype of CD226KO mice. Genotype identification of CD226KO mice was performed before the experiments (Additional file 2: Fig. S2A). After HFD feeding, the increased weight of obese mice in the KO group was significantly lower than that of WT mice (Fig. 2A, B). H&E staining and immunofluorescence staining of perilipin-1 indicated a suppressed increase of adipocyte size in the HFD CD226KO group compared with the HFD WT group (Fig. 2C, D). Additionally, the compensatory hyperplasia of islet tissue was significantly decreased in the HFD CD226KO group (Fig. 2E, F). The HFD-induced increases of serum levels of triglyceride, HDL-C, LDL-C, and total cholesterol were also relieved by CD226 deficiency (Fig. 2G).

Recent research has established that obesity is associated with chronic low-grade inflammations, contributing to an increasing prevalence of chronic metabolic diseases, such as NALFD and steatohepatitis [2]. Here, we found that the liver function parameters, including AST, ALT, γ-GT, and ALP, were decreased in the HFD CD226KO mice compared with the HFD WT group (Additional file 3: Fig. S3A). The increase of liver size and weight in HFD-fed CD226KO mice was significantly reduced compared with HFD-fed WT controls (Additional file 3: Fig. S3B, S3C). Clusters of lipid droplets detected by ORO staining were reduced in HFD CD226KO mice compared with the HFD WT group (Additional file 3: Fig. S3D). Meanwhile, H&E images of liver tissues showed that the HFD-caused pathological damages including steatosis and ballooning were also attenuated in CD226KO mice (Additional file 3: Fig. S3E). These data demonstrate that CD226KO mice are protected from HFD-induced obesity and its related liver damage.

### CD226KO suppresses ATM accumulation in mice after HFD

**Fig. 3:**
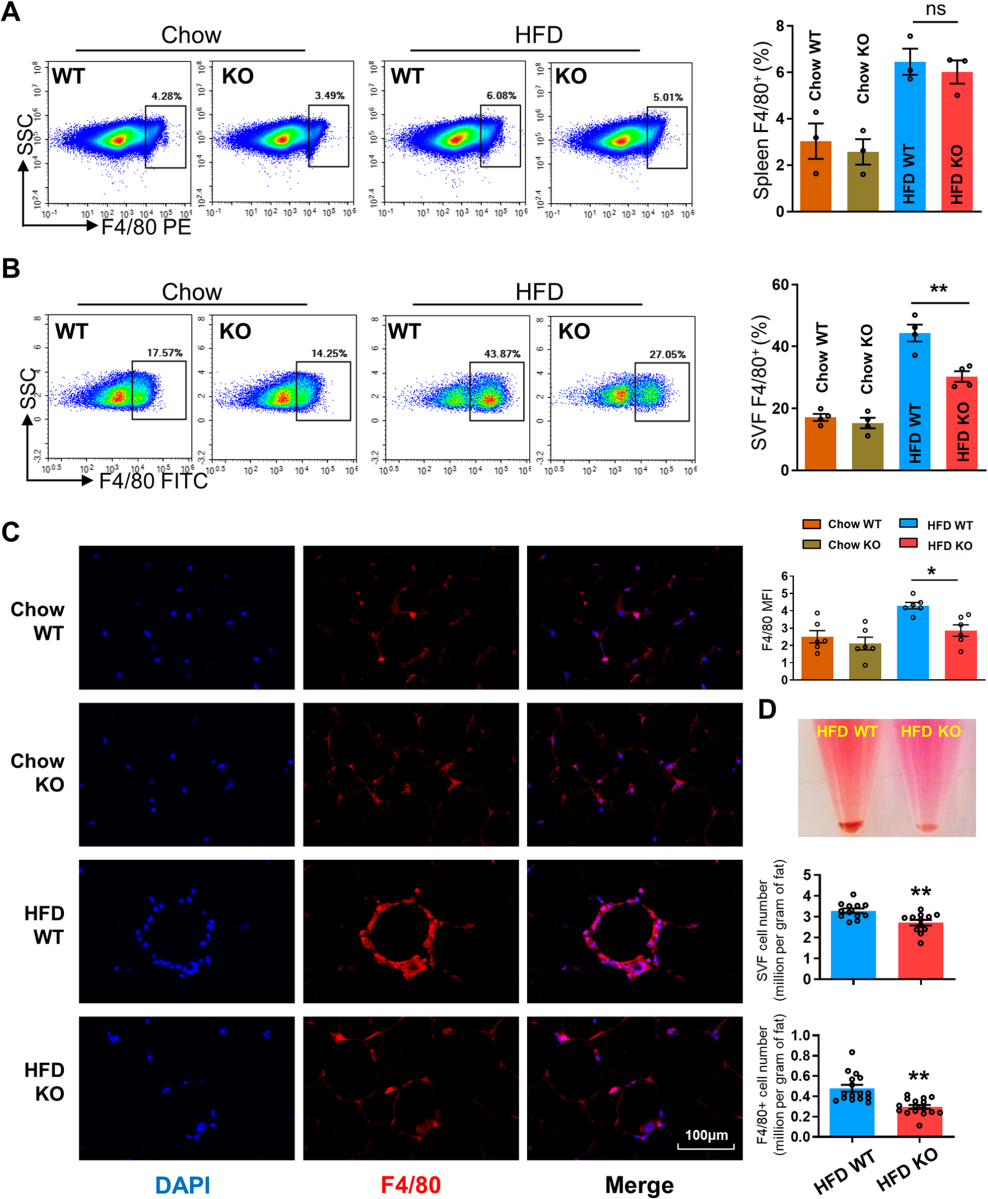
Decrease of ATMs in HFD-fed CD226KO mice. **A** FACS analysis of F4/80^+^ macrophages in the spleens of WT and CD226KO mice fed with either chow or a HFD for 16 weeks (n = 3). **B** FACS analysis of F4/80^+^ macrophages in epididymal SVF (n = 4). **C** Representative images and quantification of immunofluorescence staining of F4/80 in the epididymal fat of WT and CD226KO mice (n = 4). Scale bar means 100 μm. **D** Gross images and absolute cell count of epididymal SVF cells and F4/80^+^ macrophages obtained from groups of mice fed with chow or a HFD (n = 4). Combined data from three independent experiments. Data represent mean ± SEM. Differences between groups were determined by one way ANOVA with Tukey’s multiple test (**A**, **B**) or unpaired Student’s *t*-test (**D**). *P < 0.05, **P < 0.01

In obese patients and mice, accumulation of ATMs, especially inflammatory ATMs, is significantly increased, contributing to the inflammation of adipose tissues [20, 21]. We detected the accumulation and inflammatory phenotype of ATMs in visceral fat. In the spleen, the percentages of F4/80 + macrophages were increased in the HFD-fed mice compared with chow-fed group, but CD226KO did not alter the macrophage amount (Fig. 3A). However, HFD led to markedly increased macrophages in epididymal SVF samples, which was significantly alleviated by further CD226KO (Fig. 3B). Nevertheless, following CD226KO had no significant effect on the proportion of CD4^+^, CD8^+^ and NK cells in white adipose tissues after HFD (Additional file 4: Fig. S4A). Using immunofluorescence staining, crown-like structures could be observed in epididymal adipose tissue sections from HFD-fed mice. In HFD-fed CD226KO mice, F4/80^+^ cells and the crown-like structures were reduced (Fig. 3C). The absolute counts of isolated SVF cells and F4/80 + ATMs in the HFD CD226KO group were also lower than HFD WT (Fig. 3D). These results suggest that CD226 is required for obesity-induced ATM accumulation.

### CD226KO inhibits the M1 proinflammation phenotype of ATMs after HFD

**Fig. 4:**
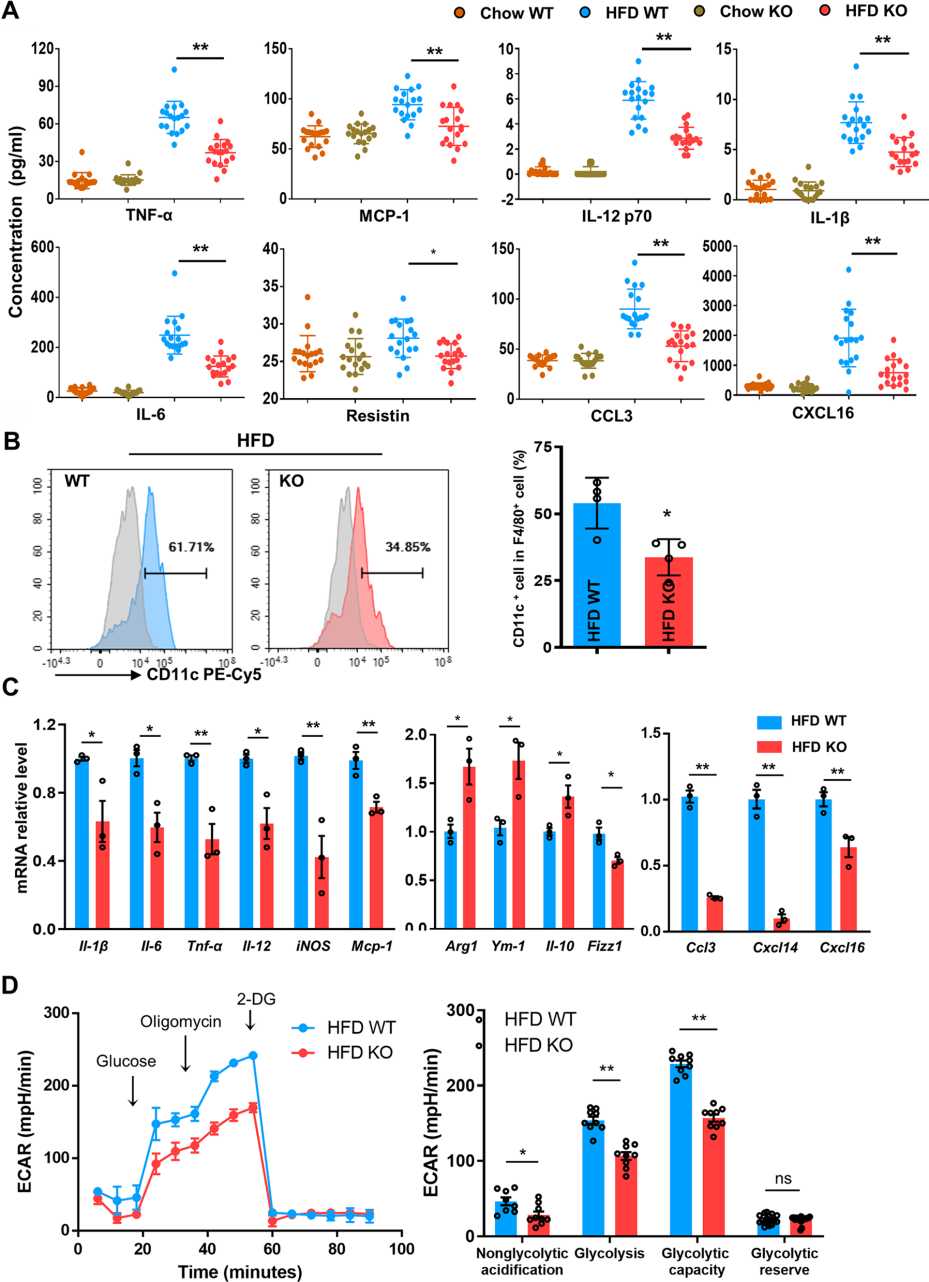
The proinflammatory ATM phenotype was reduced in HFD-fed CD226KO mice. **A** Serum concentrations of M1-type cytokines and chemokines in WT and CD226KO mice fed with chow or a HFD for 16 weeks (n = 6). **B** FACS analysis of M1-type (CD11c^+^) ATMs from WT and CD226KO mice with a HFD (n = 4). **C** mRNA levels of M1/M2-type markers and chemokines in epididymal ATMs from HFD WT and CD226KO mice (n = 3). **D** Glycolytic activity of ATMs from HFD WT and CD226KO mice (n = 3) Combined data from three independent experiments. 2-DG, 2-deoxyglucose. Data represent mean ± SEM. Differences between groups were determined by one way ANOVA with Tukey’s multiple test (**A**, **C**, **D**) or unpaired Student’s *t*-test (**B**). *P < 0.05, **P < 0.01

Given that CD226 deficiency improved obesity and its related systemic inflammation, we investigated the inflammatory phenotype of the accumulated ATMs. Luminex analysis showed that in the HFD-induced obesity mouse model, there was an obvious increase in serum proinflammation cytokines such as TNF-α, MCP-1, IL-12, IL-1β, IL-6, CCL3, CXCL16, and Resistin, which were further alleviated significantly in the CD226KO group (Fig. 4A). Furthermore, the CD11c^+^ percentage in epididymal ATMs (M1-type) decreased in HFD-fed CD226KO mice compared with the HFD-fed WT (Fig. 4B). We then isolated and cultured ATMs from the epididymal adipose tissue of HFD-fed mice for ex vivo experiments and analyzed the culture supernatant. The ATMs derived from CD226KO mice produced fewer proinflammatory cytokines than those from WT mice, both at the protein level as detected by Luminex analysis (Additional file 5: Fig. S5A) and at the mRNA level as detected by qPCR (Fig. 4C). These results showed that M1 markers or proinflammatory cytokines in the SVF of HFD-fed obese mice were lower in the CD226KO group, whereas M2 markers were higher, indicating that the proinflammatory ATM state and M1 polarization were suppressed in obese CD226KO mice.

Diverse traits characterize the phenotypes and bioenergetics of macrophages in different states. It is accepted that inflammatory M1 macrophages are more likely to depend on aerobic glycolysis to support their functions. Reportedly, aerobic glycolysis was significantly increased in SVF and ATMs isolated from obese mice compared with those isolated from lean mice [5]. In the present study, functional metabolic analysis was performed to assess the glycolysis rates of epididymal ATMs from WT and CD226KO mice fed with HFD. We found that the glycolysis capacity (i.e., real-time extracellular acidification rate, ECAR) of the ATMs was limited in the HFD CD226KO group (Fig. 4D). This result is consistent with the previous conclusion that M1-type ATMs are decreased in obese CD226KO mice.

**Fig. 5:**
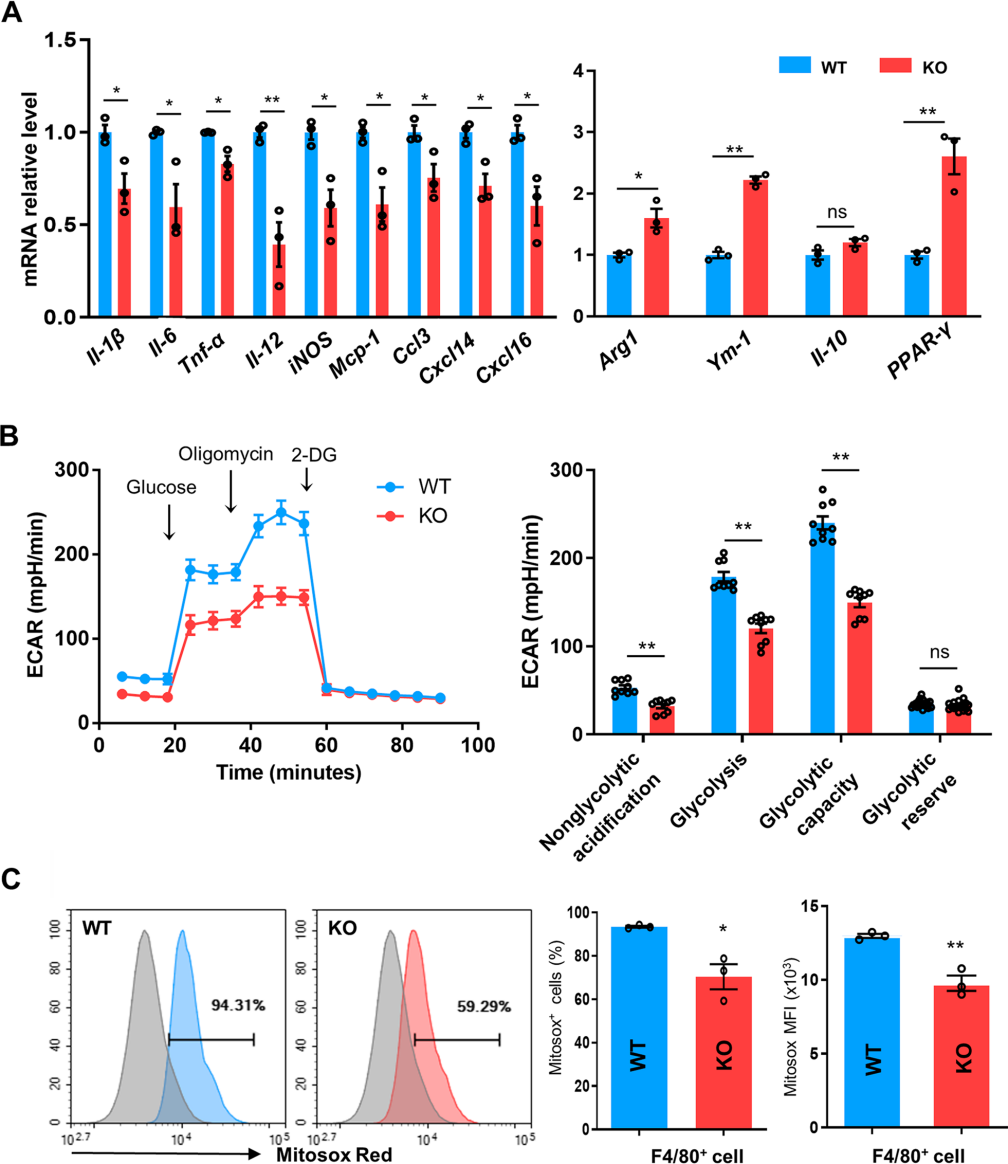
Deficiency of CD226 regulated macrophage M1/M2 polarization. **A** mRNA levels of M1/M2-type cytokines, *Ccl3*, *Cxcl14*, *Cxcl16*, and *PPAR-γ* in LPS-stimulated peritoneal macrophages from WT and CD226KO mice (n = 3). **B** Glycolytic activity of LPS-stimulated peritoneal macrophages from WT and CD226KO mice. Combined data from three independent experiments (n = 3). **C** ROS production in LPS-stimulated peritoneal macrophages from WT and CD226KO mice (n = 3). Data represent mean ± SEM. Differences between groups were determined by unpaired Student’s *t*-test. *P < 0.05, **P < 0.01

To mimic the inflammatory state of common macrophages after HFD, peritoneal macrophages from WT and CD226KO mice were isolated and cultured for 12 h with LPS stimulation. The qPCR results showed that in CD226KO macrophages, M1-type proinflammatory markers such as *Il-1β*, *Il-6*, *Il-12*, *iNOS*, *Mcp-1*, *Tnf-α*, *Ccl3*, *Cxcl14*, and *Cxcl16* reduced significantly, while M2-related molecules, including *Arg1*, *Ym-1*, and *PPAR-γ* increased (Fig. 5A). Consistent with the ATM metabolic results, CD226KO peritoneal macrophages exhibited a lower glycolysis capacity than WT controls (Fig. 5B). Interestingly, ROS production decreased in CD226KO macrophages, as shown by staining cells with mitochondrial superoxide indicator MitoSOX, implying that the inflammatory phenotype of CD226KO macrophages was suppressed (Fig. 5C).

### CD226 regulates macrophage polarization through PPAR-γ-related signaling

**Fig. 6:**
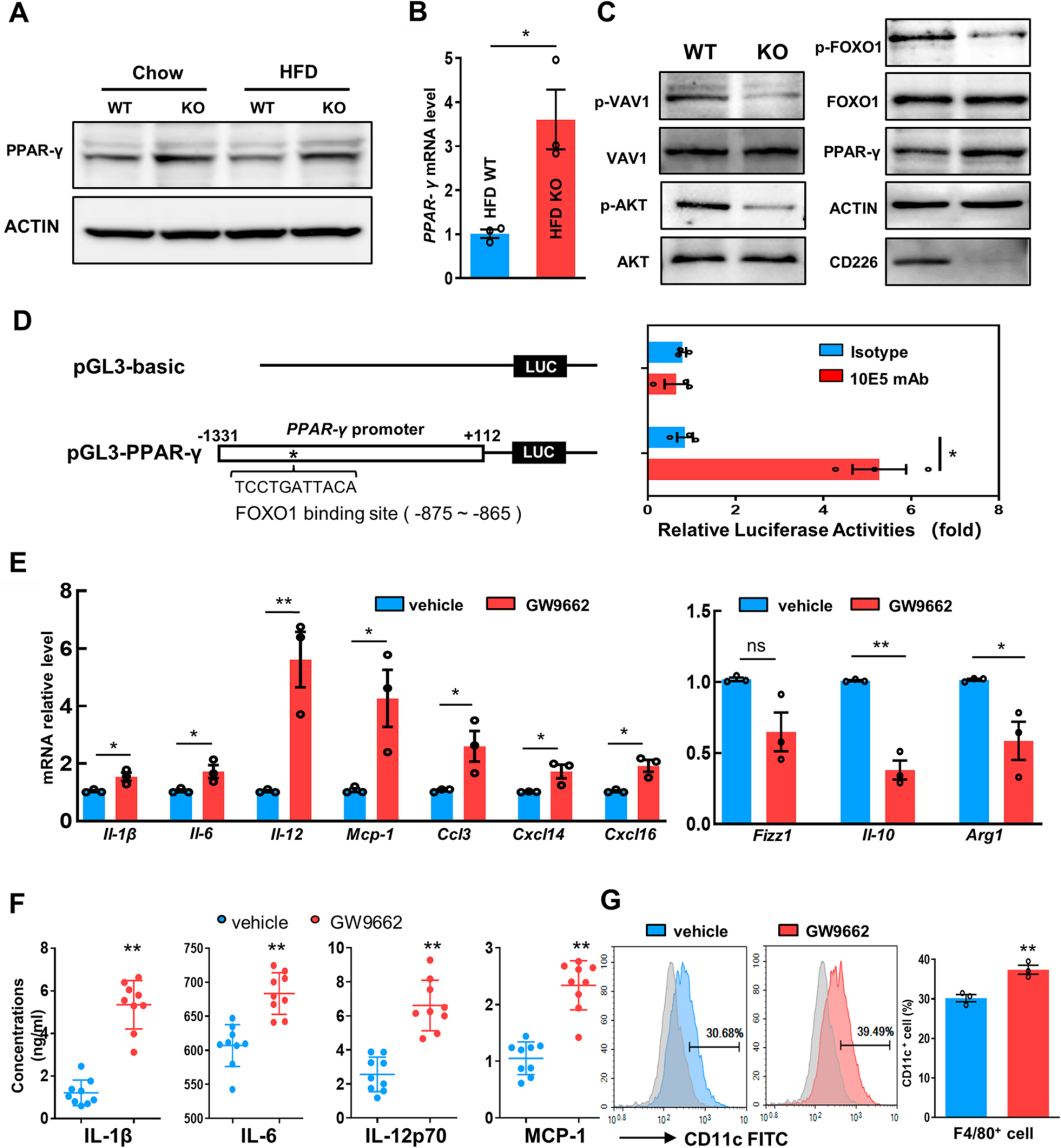
VAV1-AKT-FOXO1-PPAR-γ pathway was involved in the regulation of CD226KO on macrophage polarization. **A** Immunoblot analysis of the expression of PPAR-γ in the epididymal fat of WT and CD226KO mice fed with either chow or a HFD for 16 weeks. Blots are representative of three independent experiments. **B** mRNA levels of *PPAR-γ* in epididymal SVF from WT and CD226KO mice on a HFD (n = 3). **C** Immunoblot analysis of the expression of p-VAV1, VAV1, p-AKT, AKT, p-FOXO1, FOXO1, and PPAR-γ in peritoneal macrophages from WT or CD226KO mice. Blots are representative of three independent experiments. **D** Luciferase activities were measured in RAW264.7 cells transfected with pGL3-PPAR-γ or pGL3-basic plasmids following 10E5 or isotype antibody treatment (n = 3). **E** mRNA levels of M1/M2-type cytokines and chemokines in LPS-stimulated CD226KO peritoneal macrophages treated with the PPAR-γ specific inhibitor GW9662 or DMSO vehicle control (n = 3). **F** Culture supernatant concentrations of proinflammatory cytokines in LPS-stimulated CD226KO peritoneal macrophages treated with GW9662. Combined data from three independent experiments (n = 3). **G** FACS analysis of CD11c^+^ M1 in LPS-stimulated CD226KO peritoneal macrophages treated with GW9662 (n = 3). Data represent mean ± SEM. Differences between groups were determined by one way ANOVA with Tukey’s multiple test (**D**) or unpaired Student’s *t*-test (**B**, **E**, **F**, **G**). *P < 0.05, **P < 0.01

PPAR-γ is a critical transcription factor that is required for the alternative activation and suppression of the inflammatory macrophages [22]. We found that PPAR-γ expression was upregulated in the epididymal SVF of HFD-fed CD226KO mice compared with the WT (Fig. 6A, B). Previous studies have shown that VAV1 is responsible for CD226 signaling transduction in NK and CD4^+^ T cells [23–25]. A recent study also reported that CD226 contributes to phosphorylation-mediated Forkhead box protein O1 (FOXO1) inactivation in NK cells [26]. As AKT is responsible for phosphorylation-mediated FOXO1 inactivation, we found that CD226 deficiency suppressed the phosphorylation and activation of VAV1 and AKT (Fig. 6C), which also led to suppressed FOXO1 inactivation and degradation and further upregulated FOXO1-dependent PPAR-γ expression (Fig. 6C). Direct evidence from the dual-luciferase reporter assay additionally supports the regulation of CD226 on FOXO1/PPAR-γ signaling in RAW264.7 cells. As shown in Fig. 6D, the luciferase activity of the pGL3-PPAR-γ promoter was significantly increased following administration of CD226-specific antibody (Clone, #10E5), which indicated that CD226 blocking weakened the binding effects of FOXO1 on PPAR-γ promoter. These in vitro results support that CD226 deficiency regulates PPAR-γ activation.

To confirm this regulation, we used the PPAR-γ-specific inhibitor GW9662 to block its signal in LPS-stimulated macrophages from CD226KO mice. The previously reduced expression of proinflammatory factors in CD226KO mice (vehicle), including *Il-1β*, *Il-6*, *Il-12*, *Mcp-1*, *Ccl3*, *Cxcl14*, and *Cxcl16*, increased significantly upon PPAR-γ inhibitor treatment (Fig. 6E). Meanwhile, the increased M2-type markers such as *Fizz1*, *Il-10*, and *Arg1* was also reduced by GW9662 (Fig. 6E). The concentration of proinflammatory cytokines in the CD226KO macrophage culture supernatant increased significantly in the GW9662 group (Fig. 6F). As shown in Fig. 6G M1 polarization of CD226KO macrophages (detected by CD11c) was also elevated by the PPAR-γ inhibitor. These results confirm that the suppressive effect of CD226 deficiency on these cytokines and chemokines was dependent on PPAR-γ.

## Discussion

HFD has been shown to induce obesity and metabolic inflammation [27]. The associations between HFD and metabolic diseases, including obesity, cardiometabolic diseases, and NALFD, have attracted increasing attentions [28–30]. Recent research has established the emerging importance of immune cells in the metabolic homeostasis [31–33]. Thus, exploring the immunological mechanisms of metabolic inflammation induced by a HFD is urgently warranted. In the present study, we clarified the potential roles of CD226 in HFD-induced obesity and its related systemic inflammation.

Previous studies have shown that *Cd226* gene polymorphism is associated with the incidence of type 1 diabetes. The T allele of the *Cd226* rs763361 polymorphism is associated with a higher susceptibility to diabetes, lower onset age, and greater aggressiveness of the disease [34–37]. Our previous study showed that CD226 on endothelial cells contributes to hyperglycemia in type 2 diabetes mellitus by suppressing glucose uptake in endothelial cells[9]. This indicates potential roles of CD226 in obesity-associated metabolic diseases. However, the exact roles of CD226 in chronic inflammatory disorders have not yet been fully elucidated.

The function of CD226 on T and NK cells has been widely studied, while its role in monocytes /macrophages was relatively less reported. Among them, CD226 was studied to be involved in the monocyte adhesion to CD155-expressing cells, such as endothelial cells. Administration of CD226 antibody could block the trans-endothelial migration of monocytes over endothelial junctions [7, 38, 39]. Another study showed that the expression of CD226 on inflammatory monocytes and splenic macrophages was increased after mouse cytomegalovirus infection. CD226 on monocytes/ macrophages interacted with CD155 on NK cells to control virus infection [40]. These publications indicated that CD226 functioned as a key interactor between monocytes/macrophages and CD155-expressing cells to modulate their migration. Moreover, recent studies indicated that CD226 exerted costimulatory effects on antigen presentation mediated by small peritoneal macrophages [11]. Deletion of CD226 further improved post-infarction healing and cardiac function by favoring macrophage polarization towards reparative phenotype [10]. These results suggested that CD226 on macrophages in different tissues may play distinct roles, which needs to be specifically studied with different surroundings.

Here, we observed that CD226 was highly expressed in the obesity population and ATMs. As the most abundant class of immune cell in adipose tissue, ATMs play a crucial role in the chronic inflammation associated with obesity [33]. The infiltration of ATMs is significantly increased in obese patients [20], and macrophage accumulation is positively correlated with the severity of adipose tissue inflammation and fatty liver [41]. Additionally, the balance of M1/M2 ATMs in obese individuals is significantly weighted toward M1 [20]. Regulating the alternative activation of ATMs could modulate obesity-associated systemic inflammation and metabolic dysfunction [42, 43]. However, exact roles of CD226 on ATMs during obesity and adipose tissue inflammation remain unclear. In this study, we found that ATM accumulation and polarization toward M1 were significantly suppressed in HFD-fed CD226KO mice. Moreover, the severity of HFD-induced obesity and its related systemic inflammation was significantly improved in CD226KO mice. Through in vivo and in vitro experiments, we found that CD226-deficient ATMs exhibited suppressed M1 polarization, lower glycolysis capacity, and reduced ROS production, suggesting that CD226 deficiency inhibited ATM polarization towards proinflammatory phenotype.

Transcription factor profiling is essential to understand the mechanisms of macrophage function and heterogeneity. PPAR-γ is a vital transcription factor with diverse functions, such as regulation of adipocyte differentiation and glucose homeostasis [44]. A previous study showed that PPAR-γ is required for alternative activation, i.e., M2 activation of macrophages [45]. In particular, activation of PPAR-γ suppresses NF-κB signaling and the inflammatory response genes, thus inhibiting the activation of M1 macrophages, and shifts the M1/M2 balance toward M2 macrophages [46, 47]. In the present study, we found that the activity of PPAR-γ was significantly increased in CD226KO ATMs, accompanied by suppressed M1 macrophage activity. Furthermore, M1 phenotype was reversed in CD226KO ATMs upon administration of PPAR-γ inhibitor.

We shed light on the molecular mechanisms of CD226 signaling transduction in macrophages and investigated why CD226 is negatively corrected with PPAR-γ expression. Previous studies have shown that VAV1 mediates the transduction of CD226 signaling in NK and CD4^+^ T cells [23–25]. VAV1 is responsible for the phosphorylation of Akt and FOXO1 in T cells [48]. Phosphorylated FOXO1 would translocate from the nucleus to the cytoplasm, where it degrades [49]. Another study found that FOXO1 is responsible for the expression of PPARγ [50]. We found that when CD226 signaling was deficient or blocked, VAV1 and AKT phosphorylation was suppressed; suppressed AKT phosphorylation led to reduced FOXO1 phosphorylation, then the inactivation and degradation of FOXO1 were inhibited, causing increased FOXO1-dependent PPAR-γ expression. The potential underlying mechanisms of ATM involvement in systematic metabolic disorders have been clarified in the present study. Bijnen et al. showed that CD11c^+^ ATMs secrete CXCL14 and CXCL16 to recruit neutrophils and macrophages in the liver, which ultimately contributes to the development of fatty liver [21]. Moreover, ATM-secreted CCL3 is positively associated with the histological severity of fatty liver [1]. PPARγ has been found to attenuate the production and secretion of these chemokine receptors/ligands [51–54]. Combined with these literatures, the elevated PPAR-γ activation caused by CD226 deficiency may also partially contribute to the ameliorated obesity-related fatty liver through suppression of chemokines.

**Fig. 7:**
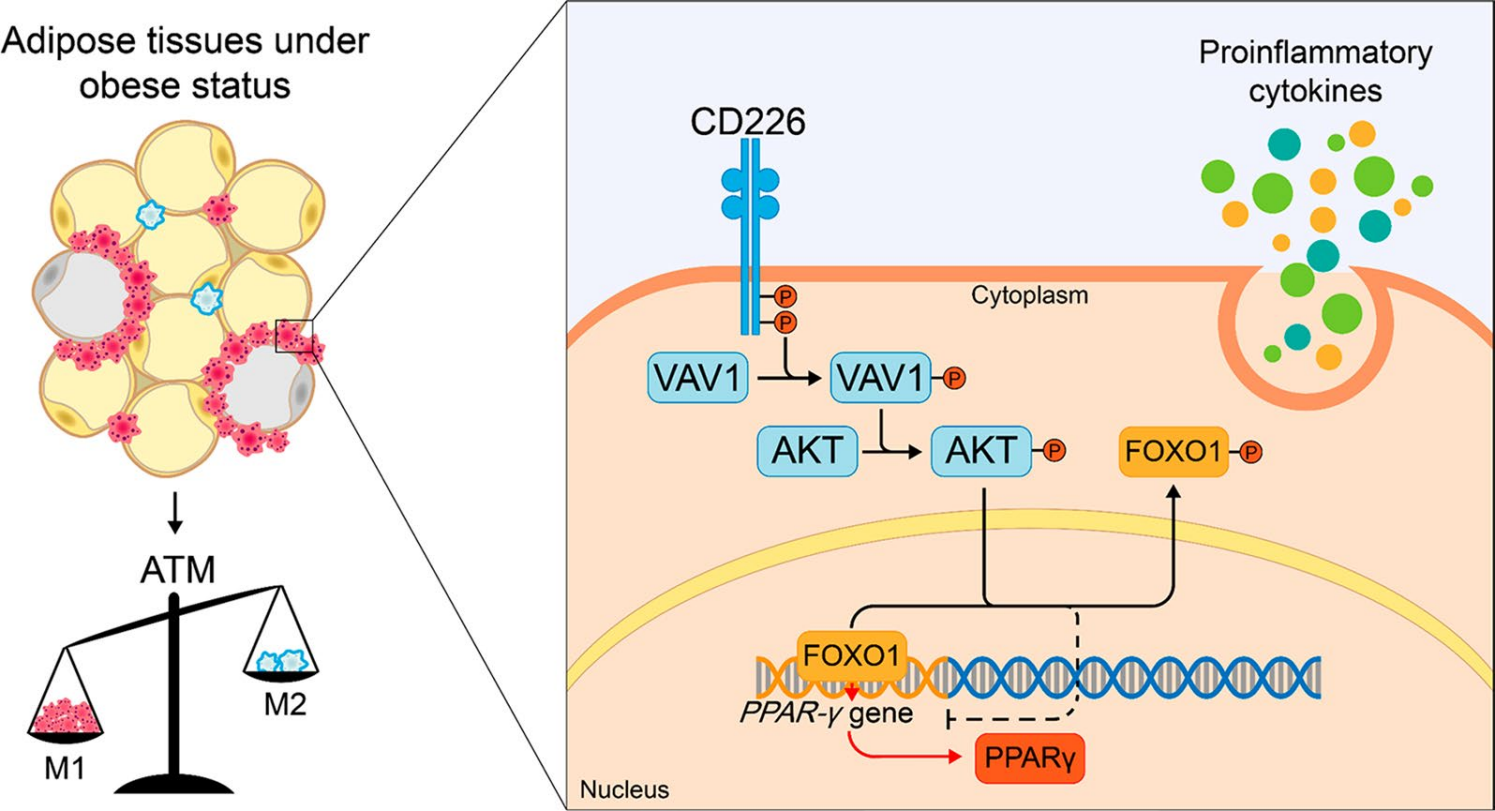
CD226 modulates the ATM polarization under a model of HFD-induced obesity. CD226 affects macrophage accumulation in adipose tissues and mediates macrophage M1 polarization. Proinflammatory M1-type ATMs induce the development of inflammation within adipose tissue and contribute to systemic inflammatory state, including fatty liver, by secreting proinflammatory cytokines through PPAR-γ-dependent signaling, which leads to the occurrence and progress of obesity

In conclusion, we proposed that CD226 deficiency could alleviate the severity of HFD-induced obesity via inhibition of the accumulation and M1 polarization of ATMs and secretion of proinflammatory cytokines. We also clarified that PPAR-γ-dependent signaling pathway is involved in the CD226-affected ATM activation (Fig. 7). Combined with the highly expressed CD226 in the obesity patients, our findings support the use of anti-CD226 for the treatment of obesity and its related metabolic disorders.

## Supplementary Information

**Additional file 1:** **Figure S1.** Expression of macrophage CD226 increased under inflammatory conditions. (A) *Cd226* mRNA levels in the epididymal fat and SVF of mice fed chow or a HFD for 16 weeks. (B) FACS analysis of CD226 expression on NK, CD4+, and CD8+ cells in the epididymal SVF of mice fed chow or a HFD for 16 weeks. (C) FACS analysis of CD155 expression on macrophages in the epididymal SVF of mice fed chow or a HFD for 16 weeks. (D) FACS analysis of CD226 expression on macrophages in mouse livers. (E) FACS analysis of CD226 expression on RAW264.7 cells with or without LPS stimulation. N=3. Data represent mean ± SEM. Intergroup differences were determined by unpaired Student’s t-test. *P < 0.05, **P < 0.01.

**Additional file 2:** **Figure S2.** (A) Representative image of CD226KO mice identification by genotyping PCR.

**Additional file 3:** **Figure S3.** Obesity-related fatty liver was alleviated in HFD-fed CD226KO mice. (A) Serum concentrations of AST, ALT, γ-GT, and ALP. Combined data from three independent experiments (n = 4 mice per group). (B) Representative liver images from WT or CD226KO mice and (C) the relative weight of the livers. Combined data from three independent experiments (n = 6 mice per group). (D) Representative images of ORO staining and quantification of positive area. Graphs from three independent experiments (n = 6 mice per group). Scale bar means 200 μm. (E) Representative images of H&E staining of livers from WT and CD226KO mice. Three independent experiments were performed (n = 7 mice per group). Scale bar means 200 μm and 50 μm. All of these samples were obtained from WT and CD226KO mice fed with chow or a HFD for 16 weeks. Data represent mean ± SEM. Differences between groups were determined by one way ANOVA with Tukey’s multiple test. *P < 0.05, **P < 0.01.

**Additional file 4:** **Figure S4.** (A) In HFD-induced obesity mice, CD226KO had no significant effect on the proportion of CD4+, CD8+ and NK cells in white adipose tissues. Representative FACS images in each group were shown.

**Additional file 5: Figure S5.** (A) Concentrations of proinflammatory cytokines and chemokines in culture supernatants of HFD WT and CD226KO mice ATMs. Combined data from three independent experiments (n = 3). Data represent mean ± SEM. Intergroup differences were determined by unpaired Student’s t-test. *P < 0.05, **P < 0.01.

**Additional file 6:** **Table S1.** Basic information for clinical participants. **Table S2. **The sequences of the qPCR primers.

## Data Availability

The datasets used and/or analysed during the current study are available from the corresponding author on reasonable request.

## Abbreviations

ATM: Adipose tissue macrophages
HFD: High-fat diet
KO: Knockout
PPAR: Peroxisome proliferator-activated receptor
IQR: Interquartile range
SVF: Stromal vascular fraction
WT: Wildtype
H&E: Hematoxylin and eosin
ORO: Oil Red O
ECAR: Extracellular acidification rate
FOXO1: Forkhead box protein O1
BMI: Body mass index
mAbs: Monoclonal antibodies
HRP: Horseradish peroxidase
FBS: Fetal bovine serum
SEM: Standard error
