# Supplementary figures and images for: CD226 knockout alleviates high-fat diet induced obesity by suppressing proinflammatory macrophage phenotype
Source: J Transl Med. 2021 Nov 25;19:477. doi: 10.1186/s12967-021-03150-4 (PMC8620575; doi:10.1186/s12967-021-03150-4)

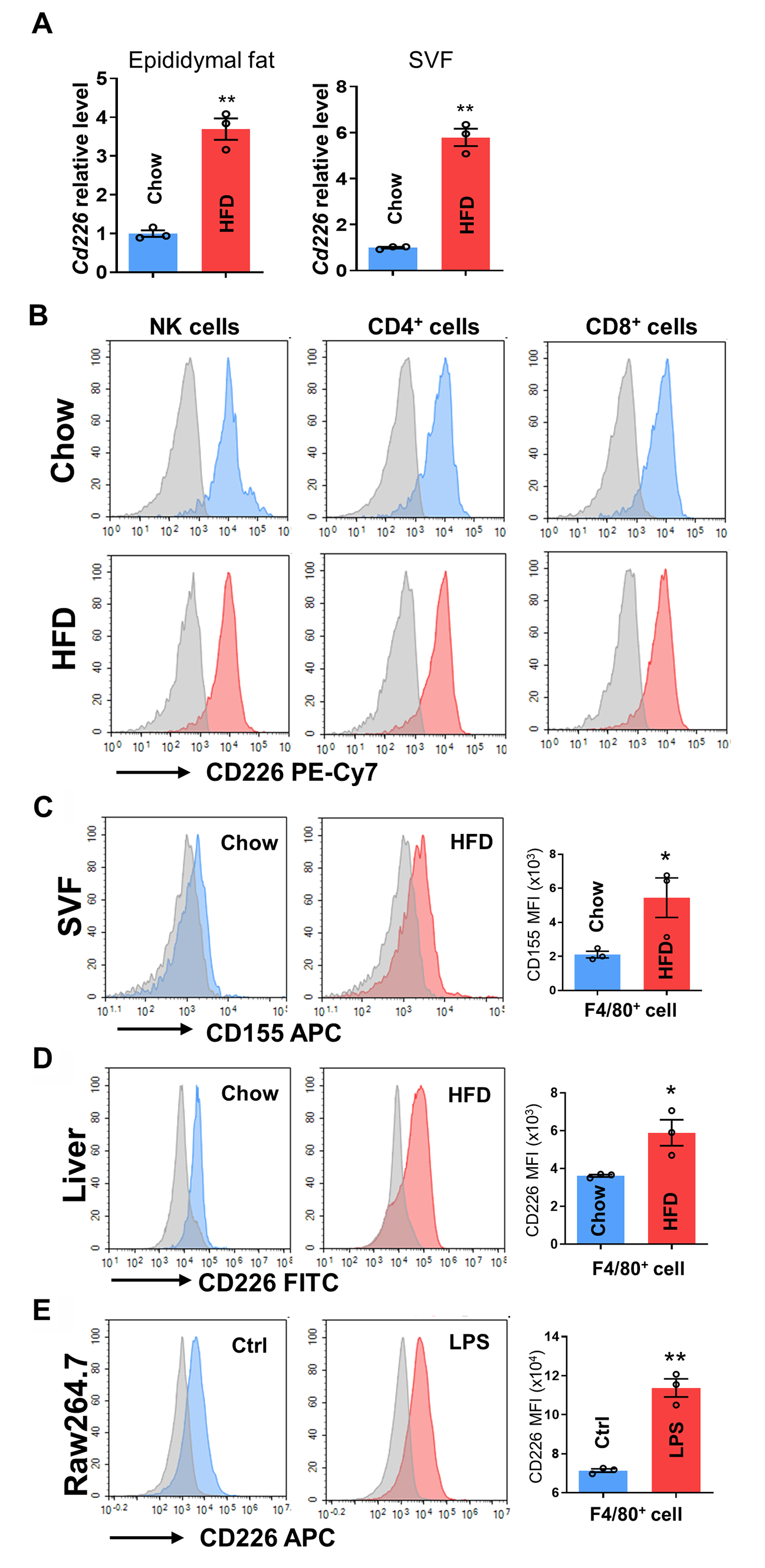

Supplement: Supplementary file 1 — Additional file 1: Figure S1. Expression of macrophage CD226 increased under inflammatory conditions. (A) Cd226 mRNA levels in the epididymal fat and SVF of mice fed chow or a HFD for 16 weeks. (B) FACS analysis of CD226 expression on NK, CD4+, and CD8+ cells in the epididymal SVF of mice fed chow or a HFD for 16 weeks. (C) FACS analysis of CD155 expression on macrophages in the epididymal SVF of mice fed chow or a HFD for 16 weeks. (D) FACS analysis of CD226 expression on macrophages in mouse livers. (E) FACS analysis of CD226 expression on RAW264.7 cells with or without LPS stimulation. N=3. Data represent mean ± SEM. Intergroup differences were determined by unpaired Student’s t-test. *P < 0.05, **P < 0.01. [file 12967_2021_3150_MOESM1_ESM.tif]

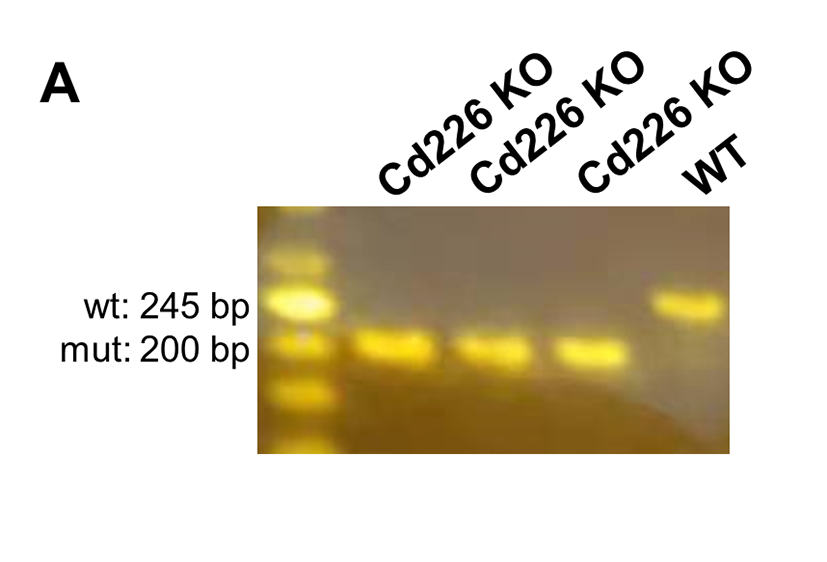

Supplement: Supplementary file 2 — Additional file 2: Figure S2. (A) Representative image of CD226KO mice identification by genotyping PCR. [file 12967_2021_3150_MOESM2_ESM.tif]

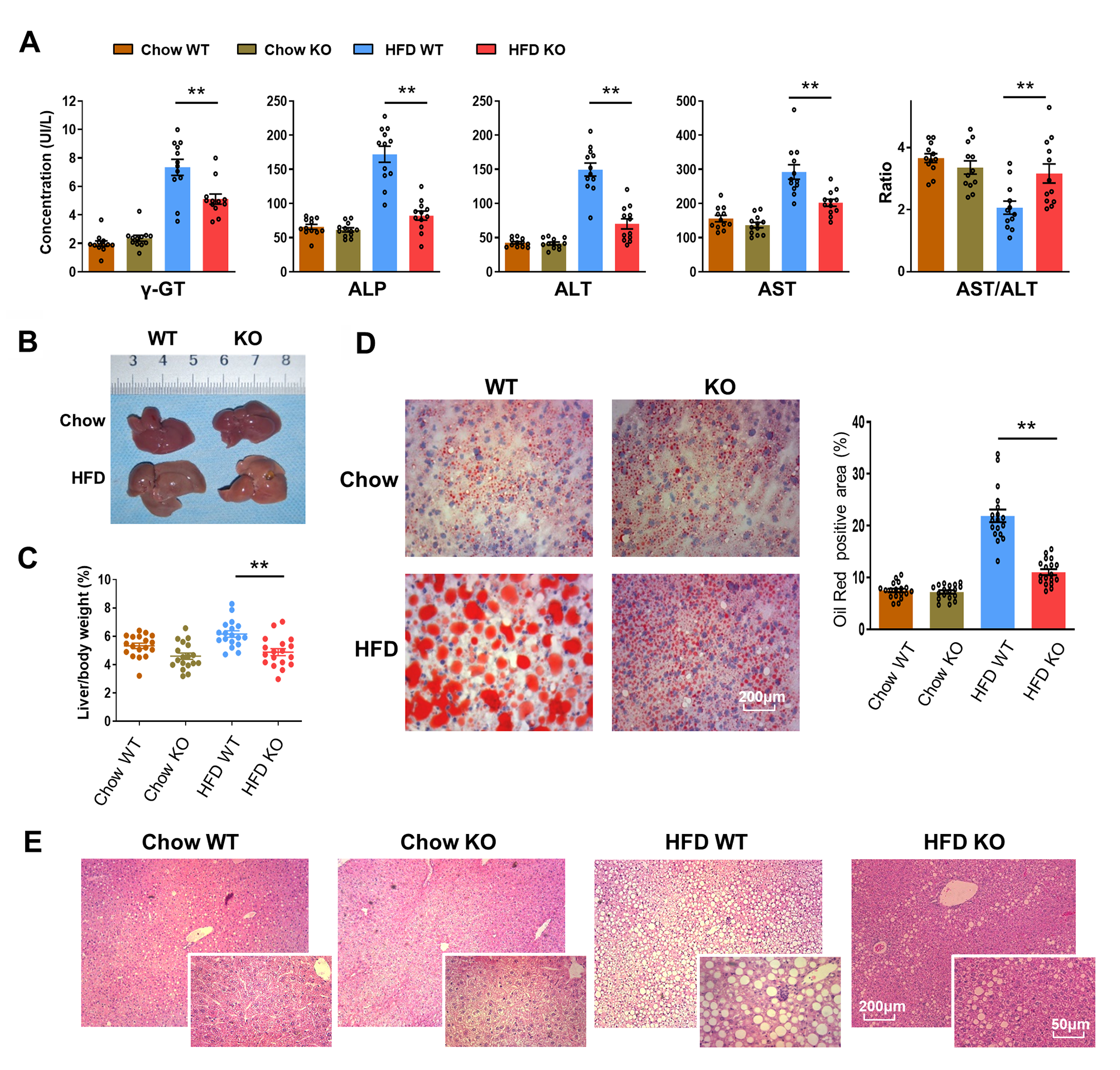

Supplement: Supplementary file 3 — Additional file 3: Figure S3. Obesity-related fatty liver was alleviated in HFD-fed CD226KO mice. (A) Serum concentrations of AST, ALT, γ-GT, and ALP. Combined data from three independent experiments (n = 4 mice per group). (B) Representative liver images from WT or CD226KO mice and (C) the relative weight of the livers. Combined data from three independent experiments (n = 6 mice per group). (D) Representative images of ORO staining and quantification of positive area. Graphs from three independent experiments (n = 6 mice per group). Scale bar means 200 μm. (E) Representative images of H&E staining of livers from WT and CD226KO mice. Three independent experiments were performed (n = 7 mice per group). Scale bar means 200 μm and 50 μm. All of these samples were obtained from WT and CD226KO mice fed with chow or a HFD for 16 weeks. Data represent mean ± SEM. Differences between groups were determined by one way ANOVA with Tukey’s multiple test. *P < 0.05, **P < 0.01. [file 12967_2021_3150_MOESM3_ESM.tif]

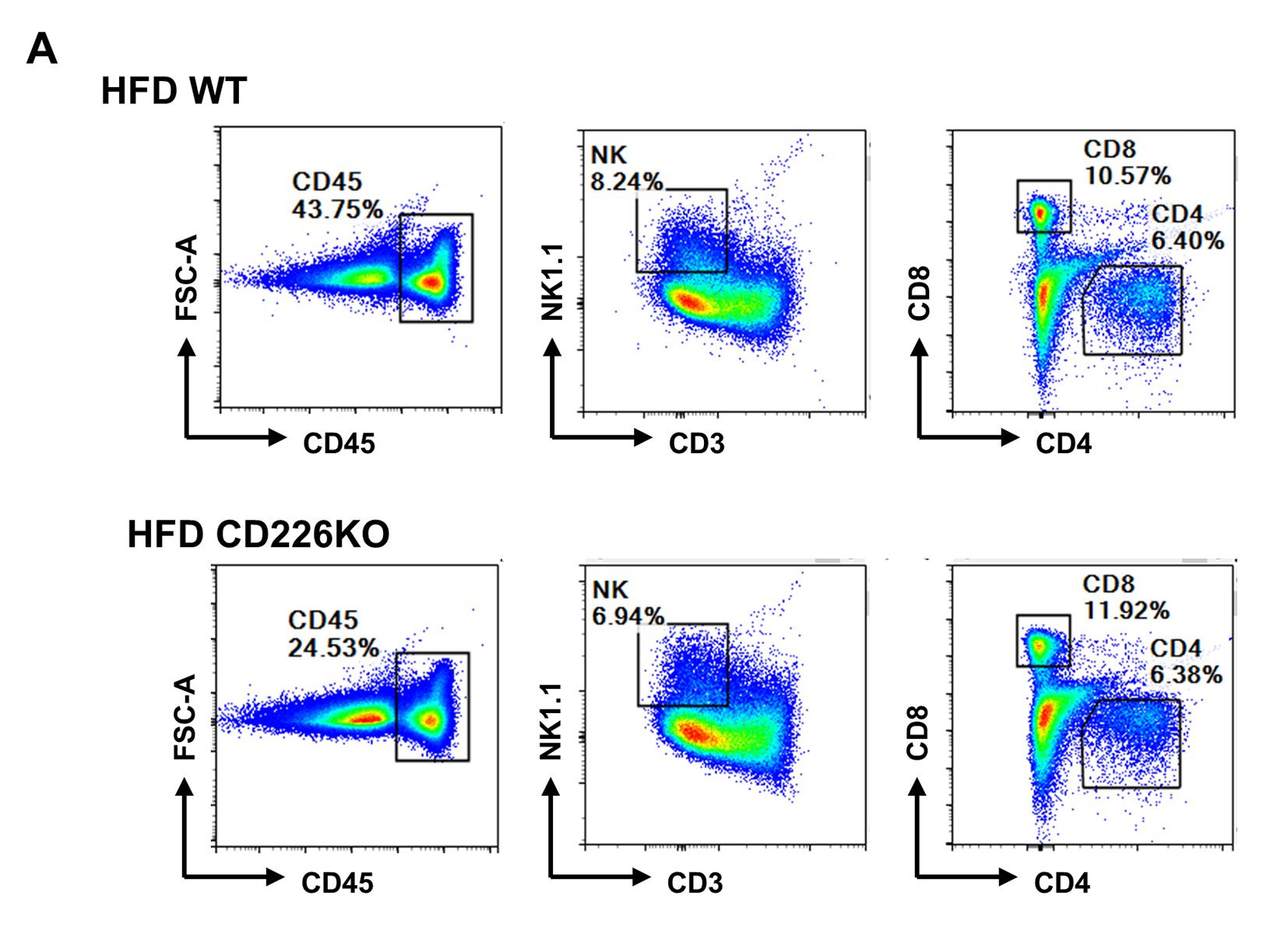

Supplement: Supplementary file 4 — Additional file 4: Figure S4. (A) In HFD-induced obesity mice, CD226KO had no significant effect on the proportion of CD4+, CD8+ and NK cells in white adipose tissues. Representative FACS images in each group were shown. [file 12967_2021_3150_MOESM4_ESM.tif]

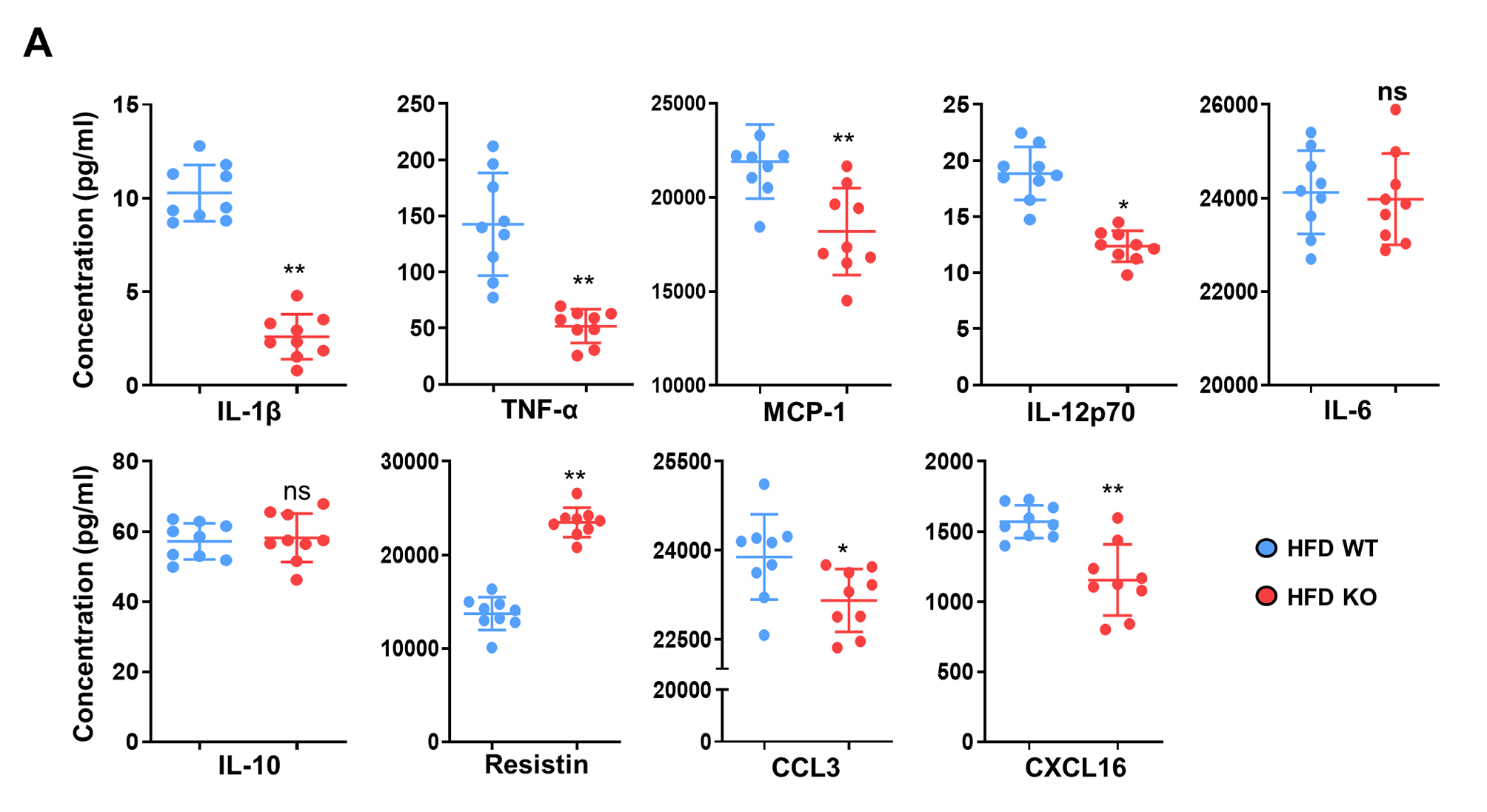

Supplement: Supplementary file 5 — Additional file 5: Figure S5. (A) Concentrations of proinflammatory cytokines and chemokines in culture supernatants of HFD WT and CD226KO mice ATMs. Combined data from three independent experiments (n = 3). Data represent mean ± SEM. Intergroup differences were determined by unpaired Student’s t-test. *P < 0.05, **P < 0.01. [file 12967_2021_3150_MOESM5_ESM.tif]
