# Supplementary material for: CD226 knockout alleviates high-fat diet induced obesity by suppressing proinflammatory macrophage phenotype
Source: J Transl Med. 2021 Nov 25;19:477. doi: 10.1186/s12967-021-03150-4 (PMC8620575; doi:10.1186/s12967-021-03150-4)
Supplement: Supplementary file 6 — Additional file 6: Table S1. Basic information for clinical participants. Table S2. The sequences of the qPCR primers. [file 12967_2021_3150_MOESM6_ESM.docx]

**Supplementary Table 1. Basic information for clinical participants**

| **Number** | **Age** | **Gender** | **Weight**  **( kg )** | **Height**  **( cm )** | **BMI**  **( kg/㎡ )** | **Number** | **Age** | **Gender** | **Weight**  **( kg )** | **Height**  **( cm )** | **BMI**  **( kg/㎡ )** |
| --- | --- | --- | --- | --- | --- | --- | --- | --- | --- | --- | --- |
| **1** | **71** | **F** | **60** | **170** | **20.76125** | **41** | **46** | **M** | **76** | **175** | **24.81633** |
| **2** | **65** | **M** | **65** | **165** | **23.87511** | **42** | **65** | **F** | **63** | **170** | **21.79931** |
| **3** | **66** | **M** | **56** | **165** | **20.56933** | **43** | **26** | **M** | **68** | **177** | **21.70513** |
| **4** | **46** | **F** | **84** | **160** | **32.8125** | **44** | **65** | **F** | **62** | **156** | **25.47666** |
| **5** | **68** | **F** | **51** | **158** | **20.42942** | **45** | **23** | **M** | **72** | **170** | **24.91349** |
| **6** | **55** | **M** | **110** | **174** | **36.33241** | **46** | **49** | **M** | **89** | **177** | **28.40818** |
| **7** | **72** | **F** | **65** | **154** | **27.40766** | **47** | **46** | **M** | **66** | **169** | **23.10843** |
| **8** | **46** | **F** | **60** | **155** | **24.97399** | **48** | **25** | **F** | **74** | **165** | **27.1809** |
| **9** | **34** | **M** | **80** | **173** | **26.72993** | **49** | **53** | **F** | **65** | **160** | **25.39063** |
| **10** | **55** | **M** | **90** | **168** | **31.88776** | **50** | **56** | **F** | **59** | **164** | **21.93635** |
| **11** | **50** | **M** | **65** | **170** | **22.49135** | **51** | **71** | **F** | **62** | **155** | **25.80645** |
| **12** | **62** | **F** | **65** | **160** | **25.39063** | **52** | **53** | **F** | **65** | **162** | **24.76757** |
| **13** | **58** | **F** | **78** | **155** | **32.46618** | **53** | **11** | **F** | **51** | **156** | **20.95661** |
| **14** | **78** | **M** | **75** | **176** | **24.21229** | **54** | **51** | **M** | **81** | **176** | **26.14928** |
| **15** | **56** | **M** | **96** | **178** | **30.2992** | **55** | **71** | **F** | **62** | **155** | **25.80645** |
| **16** | **78** | **M** | **77** | **160** | **30.07813** | **56** | **55** | **M** | **74** | **164** | **27.51338** |
| **17** | **82** | **F** | **64** | **166** | **23.22543** | **57** | **49** | **M** | **89** | **177** | **28.40818** |
| **18** | **67** | **F** | **52** | **156** | **21.36752** | **58** | **68** | **F** | **51** | **157** | **20.69049** |
| **19** | **43** | **M** | **95** | **178** | **29.98359** | **59** | **69** | **F** | **61** | **163** | **22.95909** |
| **20** | **35** | **F** | **55** | **167** | **19.72104** | **60** | **53** | **F** | **65** | **162** | **24.76757** |
| **21** | **75** | **F** | **63** | **152** | **27.26801** | **61** | **51** | **F** | **51** | **162** | **19.43301** |
| **22** | **53** | **F** | **60** | **160** | **23.4375** | **62** | **46** | **F** | **71** | **158** | **28.44095** |
| **23** | **23** | **F** | **61** | **163** | **22.95909** | **63** | **84** | **F** | **55** | **155** | **22.89282** |
| **24** | **39** | **F** | **62.5** | **173** | **20.88276** | **64** | **66** | **M** | **73** | **157** | **29.61581** |
| **25** | **67** | **M** | **88** | **172** | **29.74581** | **65** | **56** | **F** | **57** | **157** | **23.12467** |
| **26** | **19** | **M** | **70** | **175** | **22.85714** | **66** | **76** | **F** | **66** | **162** | **25.14861** |
| **27** | **49** | **M** | **76** | **175** | **24.81633** | **67** | **65** | **F** | **61** | **152** | **26.40235** |
| **28** | **81** | **F** | **59** | **152** | **25.5367** | **68** | **68** | **F** | **51** | **157** | **20.69049** |
| **29** | **75** | **F** | **52** | **153** | **22.21368** | **69** | **69** | **F** | **61** | **163** | **22.95909** |
| **30** | **52** | **M** | **76** | **174** | **25.10239** | **70** | **51** | **F** | **51** | **162** | **19.43301** |
| **31** | **59** | **F** | **54** | **153** | **23.06805** | **71** | **42** | **F** | **48** | **159** | **18.98659** |
| **32** | **65** | **F** | **48** | **155** | **19.97919** | **72** | **68** | **M** | **72** | **168** | **25.5102** |
| **33** | **63** | **F** | **60** | **148** | **27.39226** | **73** | **74** | **F** | **44** | **154** | **18.55288** |
| **34** | **42** | **M** | **67** | **170** | **23.18339** | **74** | **55** | **M** | **74** | **164** | **27.51338** |
| **35** | **28** | **M** | **82** | **174** | **27.08416** | **75** | **18** | **F** | **51** | **164** | **18.96193** |
| **36** | **68** | **F** | **59** | **150** | **26.22222** | **76** | **66** | **M** | **73** | **157** | **29.61581** |
| **37** | **48** | **F** | **54** | **165** | **19.83471** | **77** | **68** | **F** | **51** | **157** | **20.69049** |
| **38** | **55** | **M** | **82** | **173** | **27.39818** | **78** | **11** | **F** | **51** | **156** | **20.95661** |
| **39** | **65** | **F** | **59** | **150** | **26.22222** | **79** | **72** | **M** | **76** | **170** | **26.29758** |
| **40** | **29** | **M** | **77** | **166** | **27.9431** |  |  |  |  |  |  |

**Supplementary Table 2. The sequences of the qPCR primers**

| Gene |  | Sequence |
| --- | --- | --- |
| *Actin* | Forward | CATTGCTGACAGGATGCAGAAGG |
|  | Reverse | TGCTGGAAGGTGGACAGTGAGG |
| *I1-1β* | Forward | TGGACCTTCCAGGATGAGGACA |
|  | Reverse | GTTCATCTCGGAGCCTGTAGTG |
| *Il-6* | Forward | TACCACTTCACAAGTCGGAGGC |
|  | Reverse | CTGCAAGTGCATCATCGTTGTTC |
| *Tnf-α* | Forward | GGTGCCTATGTCTCAGCCTCTT |
|  | Reverse | GCCATAGAACTGATGAGAGGGAG |
| *Il-12* | Forward | ACGAGAGTTGCCTGGCTACTAG |
|  | Reverse | CCTCATAGATGCTACCAAGGCAC |
| *iNOS* | Forward | GAGACAGGGAAGTCTGAAGCAC |
|  | Reverse | CCAGCAGTAGTTGCTCCTCTTC |
| *Mcp-1* | Forward | GCTACAAGAGGATCACCAGCAG |
|  | Reverse | GTCTGGACCCATTCCTTCTTGG |
| *Arg1* | Forward | CATTGGCTTGCGAGACGTAGAC |
|  | Reverse | GCTGAAGGTCTCTTCCATCACC |
| *Ym-1* | Forward | TACTCACTTCCACAGGAGCAGG |
|  | Reverse | CTCCAGTGTAGCCATCCTTAGG |
| *Il-10* | Forward | CGGGAAGACAATAACTGCACCC |
|  | Reverse | CGGTTAGCAGTATGTTGTCCAGC |
| *Fizz1* | Forward | CAAGGAACTTCTTGCCAATCCAG |
|  | Reverse | CCAAGATCCACAGGCAAAGCCA |
| *Ccl3* | Forward | ACTGCCTGCTGCTTCTCCTACA |
|  | Reverse | ATGACACCTGGCTGGGAGCAAA |
| *Cxcl14* | Forward | TACCCACACTGCGAGGAGAAGA |
|  | Reverse | CGCTTCTCGTTCCAGGCATTGT |
| *Cxcl16* | Forward | GCAGGGTACTTTGGATCACATCC |
|  | Reverse | AGTTCACGGACCCACTGGTCTT |
| *PPAR-γ* | Forward | GTACTGTCGGTTTCAGAAGTGCC |
|  | Reverse | ATCTCCGCCAACAGCTTCTCCT |
